# Article Commentary: Indicators to Measure Success of Smoke-free Policies

**DOI:** 10.4137/EHI.S898

**Published:** 2008-07-18

**Authors:** Amanda Niskar

**Affiliations:** Sackler Senior Faculty of Medicine, School of Public Health, Department of Environmental and Occupational Health, Tel Aviv University, Tel Aviv, Israel.

**Keywords:** Smoking, policy, indicators, evaluation, indoor air quality

## Abstract

The health consequences of involuntary exposure to tobacco smoke are well documented. The past decade provides several examples of successful smoke-free policies that are directly related to improving public health. The objective of this communication is to identify indicators that demonstrate the success of smoke-free policies. Indicators are identified from smoke-free policy evaluations conducted by countries such as the United States, New Zealand, and Israel. Indicators were identified that demonstrate the success of smoke-free policies in the areas of compliance, indoor air quality, bio-monitoring, medical tests, and health behaviors. As smoke-free policies continue to be implemented, indicator evaluation activities should be considered from the start.

## Background

The health consequences of involuntary exposure to tobacco smoke are well documented (U.S. Department of Health and Human Services 2006). Smoke-free policies have been developed and implemented at the local and national level in many countries. These policies are of significance to public health because they have been shown to directly result in improving respiratory health (Menzies et al. 2006) and to be associated with drops in acute myocardial infarction (Sargent et al. 2004) as well as other human health benefits. Smoke-free policies continue to be developed and implemented by individual cities and countries. The past decade provides several examples of successful local and national smoke-free policies. As smoke-free policies continue to be implemented, indicator evaluation activities should be considered from the start. The objective of this communication is to identify indicators that demonstrate the success of smoke-free policies in the areas of compliance, indoor air quality, bio-monitoring, medical tests, and health behaviors.

## Compliance

New York, California, and Israel have documented the following examples of compliance with smoke-free policies. Inspections conducted to assess compliance with New York City's smoke-free law found that 97 percent of restaurants and bars prohibited smoking, removed ashtrays, and posted “no smoking” signs (New York City Department of Finance et al. 2004). Within one month of the New York state smoke-free law taking effect, the proportion of restaurants, bars, and bowling facilities statewide where no smoking was observed increased from 31 percent to 93 percent (New York State Department of Health 2004). After the law was implemented, patron compliance with California's smoke-free bar law in bars increased from 46 percent to 76 percent (Weber et al. 2003). In Israel, a woman sued in small claims court for compensation for the exposure of herself and her children to tobacco smoke in a restaurant in violation of the law restricting smoking in public places (ra “a 9615/05 Shemesh v Fokacheta Ltd, 2006). After the small claims court found that the restaurant's owners were liable for nominal compensation and the district court affirmed the decision, the Supreme Court agreed to consider a further appeal. In its decision, including reference to the Framework Convention on Tobacco Control, the Supreme Court increased the compensation tenfold and also awarded the plaintiff a similar sum for expenses and lawyer's fees (Simpson, 2006).

Public support for smoke-free policies has been measured and documented in New York, California, and Israel. An evaluation of the New York state tobacco control program found that the proportion of adults who reported supporting the state's smoke-free law increased from 64 percent before the law to 79 percent after the law took effect. Support for the law among smokers increased from 25 percent before the law to 46 percent after the law (New York State Department of Health 2006). The proportion of California patrons who approved of the law increased from 46 percent to 62 percent after the state smoke-free bar law went into effect (Tang et al. 2003). A study of California's smoke-free bar law found that 51 percent of bar owners and staff working in stand-alone bars preferred to work in a smoke-free environment after the law went into effect compared to 17 percent before the law (Tang et al. 2004). In 2002, a 1983 law banning smoking in public places was expanded to ban smoking in malls and shopping centers. The Israel Cancer Association (2007) found that 75% of the Israeli public supports the prohibition against smoking in restaurants and cafes.

Smoke-free policies in workplaces such as bars and restaurants result in positive economic impacts that may influence compliance and public support for smoke-free policies (Dearlove et al. 2002). For example, studies in New Zealand and United States found that smoke-free policies in bars and restaurancts maintain or increase tax revenues and employment levels (Glantz and Smith, 1997; Scollo et al. 2003; Dai et al. 2004; Huang et al. 2004; New York City Department of Finance et al. 2004; Cowling and Bond, 2005; Thomson and Wilson, 2006). The studies described in this section demonstrate compliance of smoke-free policies, but these studies are difficult to compare due to a lack of standard protocols to assess compliance of smoke-free policies in public settings. For example, it would be helpful to develop standards for indicators of compliance such as frequency of measurement and duration of measurement.

## Indoor Air Quality

Ireland, Boston, and New York have monitored the indoor air quality parameters associated with smoking to determine if the smoke-free policies improved indoor air quality. A total workplace smoking ban in Ireland resulted in an 83% reduction of indoor particulate matter (PM2.5) and an 80.2% reduction in benzene concentrations in the bars (Goodman et al. 2007). In addition, there was an 83% reduction in air nicotine concentrations in the bars (Mulcahy et al. 2005). A smoking ban in Boston brought a 90% to 95% reduction in polycyclic aromatic hydrocarbons and respirable suspended particles (Repace et al. 2006). New York implemented a state law requiring indoor workplaces and public places to be smoke-free resulting in average levels of respirable suspended particles decreasing by 84 percent at these indoor locations (Centers for Disease Control and Prevention 2004). A Surgeon General Report provides more discussion about the methods and results of air monitoring (U.S. Department of Health and Human Services 2006). The examples provided in this section are typical.

## Bio-Monitoring and Medical Tests

Ireland and New York conducted bio-monitoring to evaluate the impact of smoke-free policies. Cotinine is a biomarker of recent passive smoking exposure that is produced when the body breaks down nicotine absorbed over the previous 2-3 days. After an Irish indoor workplace smoking ban, a study reported saliva cotinine concentrations among hospitality staff reduced by 69% (Mulcahy et al. 2005). Another study in Ireland found an 81% reduction in salivary cotinine, a 79% reduction in exhaled breath carbon monoxide, and statistically significant improvements in lung function among non-smoking barmen after the smoking ban (Goodman et al. 2007). A study in New York found employees’ cotinine levels decreased by 78 percent after the New York state smoke-free law took effect (Farrelly et al. 2005). In addition, an adult survey sample in New York found saliva cotinine levels fell by about 50 percent (New York State Department of Health, 2004).

## Health Behaviors

This section highlights the importance of the synergy of policy efforts, media or education campaign, and population-based cessation strategies. The United States has a few studies that measured changes in health behaviors related to smoking that are associated with the implementation of smoke-free policies. Among New York City adults, smoking prevalence decreased by 11% from 2002 to 2003 following the implementation of a smoke-free city law, a cigarette excise tax increase, a media campaign, and a cessation initiative involving the distribution of free nicotine replacement therapy (Frieden et al. 2005). A U.S. national study found that adolescents who work in smoke-free workplaces are significantly less likely to be smokers than adolescents who work in workplaces without smoking restrictions (Farkas et al. 2000). A Massachusetts study found that youth living in towns with smoke-free restaurants were less than half as likely to establish smoking compared to youth living in towns with restaurants that are not smoke-free (Siegel et al. 2005).

In California, tobacco control policies were examined utilizing a simulation model (Levy et al. 2007). Policies regarding taxes, mass media, clean air laws, and youth access policies were modeled independently and as a package. Results showed that tobacco control policies reduced smoking rates in California by an additional 25% relative to the level that they would have been if policies were kept at their 1988 level. By 2004, the model attributed 59% of the reduction in smoking rates to price increases, 28% of the overall effect to media policies, and 11% to clean air laws.

## Conclusions

In summary, the published literature evaluating the impact of smoke-free policies has used many indicators. Indicators of compliance include removing ashtrays, no smoking signs, and public support for the law. Indicators of indoor air quality include indoor pm 2.5, benzene, air nicotine, polycyclic aromatic hydrocarbons and respirable suspended particles. Bio-monitoring indicators include saliva cotinine levels and exhaled breath carbon monoxide. In addition, medical tests include lung function tests. Changes in behavior can be monitored with indicators regarding initiation of smoking among adolescents and adults and smoking cessation among current smokers.

Smoke-free policies implemented on the local and national level in the past decade have many success stories for improving economics at the local workplaces, improving compliance and public support, reducing indoor air pollution while raising indoor air quality and reducing smoking biomarkers in people. Changes in health behaviors among the youth are noted too. There are more cities and countries implementing smoke-free policies, and it would be helpful if these policies are evaluated for their impact by these indicators of compliance, indoor air quality, bio-monitoring, medical testing, and health behavior. In addition, researchers are exploring indicators and methodologies to measure smoke-free policies beyond indoor public places; including areas such as outdoor dining, public parks, personal vehicles, multi-unit housing, and other locations. The Centers for Disease Control and Prevention (2008) recently published a toolkit that provides guidance for how to assess the impact of smoke-free policies.

## Disclaimer

The manuscript does not necessarily reflect the views of the Israel Ministry of Health.
